# Systemic Alterations of Wnt Inhibitors in Patients with Prostate Cancer and Bone Metastases

**DOI:** 10.1155/2018/1874598

**Published:** 2018-07-18

**Authors:** Stefan Aufderklamm, Jörg Hennenlotter, Phillip Leidenberger, Steffen Rausch, Andrea Hohneder, Ursula Kühs, Moritz Maas, Christian Schwentner, Jens Bedke, Arnulf Stenzl, Tilman Todenhöfer

**Affiliations:** ^1^Department of Urology, Eberhard Karls University of Tübingen, Tübingen, Germany; ^2^Department of Urology, Diakonie-Klinikum Stuttgart, Stuttgart, Germany

## Abstract

**Purpose:**

Dickkopf-1 (DKK-1) and sclerostin seem to inhibit osteoblast activity by blocking the Wnt pathway, which leads to progression of metastatic prostate cancer (PC). However, it is unknown whether serum levels of these proteins are altered in PC patients with or without metastasis. The aim of this study was to assess DKK-1 and sclerostin serum levels in PC patients, including patients with bone metastases.

**Methods:**

The study cohort (*N* = 143) consisted of 53 controls with benign prostatic hyperplasia (BPH), 43 with localized PC (PC cM0), and 47 had PC with metastasis (PC cM1). Serum levels of DKK-1 and sclerostin were measured by enzyme-linked immunosorbent assay. Results were compared using the Kruskal-Wallis tests; post hoc analysis was performed using the Tukey-Kramer test.

**Results:**

Mean DKK-1 levels in patients with BPH (2809.4 pg/ml) (*p* < 0.001) as well as PC cM1 (2575.5 pg/ml) (*p* = 0.001) were significantly higher than in patients with PC cN0 cM0 (1551.8 pg/ml). Among PC cM1 patients, median DKK-1 levels were significantly lower in patients with castration-resistant disease compared to those with hormone-sensitive PC (*p* = 0.02); in contrast, sclerostin concentrations were elevated (*p* = 0.04). DKK-1 correlated with PSA in the cM1 group (*p* = 0.03) and sclerostin correlated with PSA in the PC group (0.01).

**Conclusions:**

DKK-1 is involved in the progression of PC. DKK-1-mediated inhibition of osteoblasts, which contributes to tumor progression and osteolytic metastases, may also play a role in the development of metastases with osteoblastic features. The use of DKK-1 antibodies should be considered for studies including metastatic PC patients.

## 1. Introduction

Prostate cancer (PC) is the most common cancer in elderly men in western countries [1]. Patients with advanced PC commonly develop bone metastases [2]. Metastatic bone disease provokes loss of bone mass, and patients may suffer from pathological fractures and other skeletal-related events (SREs) [3]. Recently, the role of the Wnt pathway in progression of osteolytic disease has been investigated [4]. In this context, antagonistic proteins Dickkopf-1 (DKK-1) and sclerostin were highlighted because they both inhibit osteoblast activity by blocking Wnt pathway signaling [4]. In other malignancies, such as multiple myeloma or breast cancer, higher levels of DKK-1 were associated with increased bone lesions [5, 6].

Expression of DKK-1 in PC tissue has been demonstrated to be elevated compared with benign tissue and appears to be associated with worse survival [7]. By contrast, downregulation of DKK-1 seems to delay the development of bone metastases in PC [8]. Furthermore, elevated DKK-1 expression is an early event in PC and with tumor progression DKK-1 expression declines, particularly in advanced bone metastases.

The Wnt pathway inhibitor sclerostin has been also discussed as a therapeutic target for cancer-related bone disease. In men with PC undergoing antihormonal treatment, circulating sclerostin levels are elevated. However, the role of sclerostin in progression of malignant bone disease is largely unknown [9].

The aim of this study was to assess systemic alterations of DKK-1 and sclerostin in patients with different stages of PC. Moreover, we assessed serum levels in patients with benign prostatic hyperplasia (BPH).

## 2. Material and Methods

### 2.1. Patients' Assessment

A total of 143 patients were included in this study. Serum samples were obtained between 2011 and 2013 after receiving informed consent from patients and approval of the institutional review board of the Eberhard Karls University of Tübingen (number 034/2011BO2 and 113/2012BO2). Bone scintigraphy was performed in all patients with PC. We included 53 patients with BPH prior to transurethral resection of the prostate (TUR-P), 43 patients with histologically proven PC before radical prostatectomy, and 47 patients with metastatic PC. Serum levels of DKK-1 and sclerostin were measured by enzyme-linked immunosorbent assay (ELISA).

### 2.2. Measurements of DKK-1 and Sclerostin

Serum samples were stored at −80°C until use. ELISA kits for both DKK-1 and sclerostin were provided by Biomedica (Vienna, Austria), and analysis was performed according to the manufacturer's protocol.

Briefly, for DKK-1, 20 *μ*l of serum was incubated with 50 *μ*l of biotinylated DKK-1 antibody for 2 hours at room temperature. After repeated washing steps with wash buffer, 100 *μ*l of conjugate was added into each well of a 96-well plate and incubated for 1 hour at room temperature. Another washing followed before 100 *μ*l of substrate was added and incubated at room temperature.

For sclerostin, 20 *μ*l of serum was incubated with 50 *μ*l of biotinylated antisclerostin antibody for 18–24 hours at room temperature. After washing with 300 *μ*l diluted wash buffer, 200 *μ*l of conjugate was added to each well and incubated for 1 hour at room temperature in the dark. Thereafter, another washing step followed and 200 *μ*l of substrate were added and incubated for 30 minutes at room temperature. Finally, 50 *μ*l of stop solution was added to each well and absorbance was measured immediately at 450 nm with reference at 630 nm. An Anthos 2010 system (Anthos Mikrosysteme GmbH, Krefeld, Germany) was used for all measurements.

### 2.3. Statistical Analysis

Serum concentrations were compared among different clinical groups (BPH, PC cM0, and PC cM1). Planned subgroup analysis included PC M1 patients with castration-sensitive (CS) and castration-resistant (CR) disease. Associations between groups were assessed by Kruskal-Wallis tests, with the Tukey-Kramer method used in post hoc analyses. Correlations between continuous variables were performed with linear regression analyses. JMP 7.0® software was used, and *p* values *p* < 0.05 were considered significant.

## 3. Results

### 3.1. Patients

Patient characteristics and clinicopathological features of defined subgroups of PC (cM0 and cM1) are summarized in Table 1. Median ages of all patients, patients with benign prostate histology, PC cM0, and PC cM1 were 73 (range 44–89), 74 (44–89), 69 (48–85), and 74 years (52–84), respectively.

Of 43 cM0 patients with radical prostatectomy, 41 (95.3%) were pN0 and 2 were pN1 (4.7%). Median PSA was 6.96 ng/ml (range 0.31–44 ng/ml). The median Gleason Score was 7 (Table 1).

In the PC cM1 group, all 47 patients had radiologic evidence of bone metastases. In addition, 25 (53.2%) patients were cN1 and 8 (17.0%) were cM1c. Lymph node status could not be assessed in two (4.3%) patients. The median PSA value in PC cM1 patients was 80.4 ng/ml (range 0.5–674.0 ng/ml). The median Gleason Score was 9. In the PC M1 group, 34 patients (72.3%) were castration-resistant (CR) and 11 patients (23.4%) showed ongoing response to antihormonal treatment; this information was unavailable for the other two (4.3%) patients (Table 1).

### 3.2. DKK-1 Serum Concentrations

**Figure 1:**
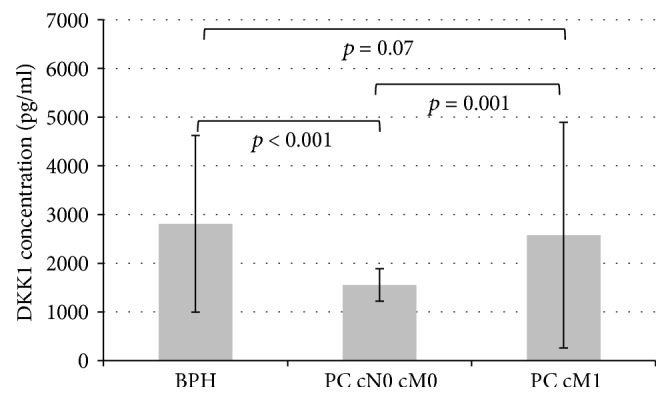
Mean DKK-1 concentrations with standard error of the mean (SEM) in patients with BPH, prostate cancer without metastases (PC cM0), or prostate cancer with metastases (PC cM1).

Mean serum concentrations of DKK-1 in patients with BPH, PC cM0, and PC cM1 were 2809.4 pg/ml (SEM 249.4), 1551.8 pg/ml (SEM 51.9), and 2575.5 pg/ml (SEM 341.7). DKK-1 levels in PC cM1 patients were significantly higher than in PC cM0 patients (*p* = 0.001). Furthermore, DKK-1 levels in patients with BPH were higher compared to patients with PC cM0 (*p* < 0.001), but were not significantly different from those with PC cM1 (*p* = 0.07; Figure 1).

**Figure 2:**
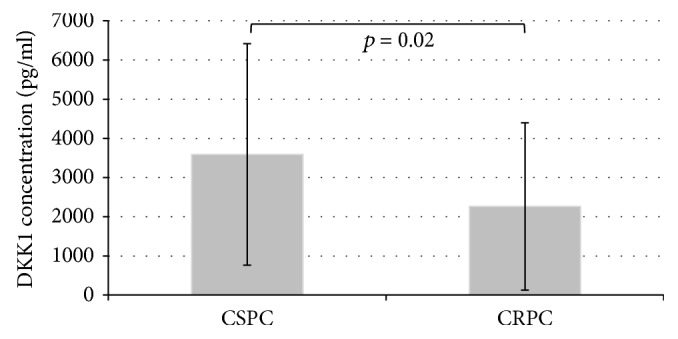
Mean serum Dickkopf-1 (DKK-1) concentrations with standard error of the mean (SEM) in patients with castration-sensitive prostate cancer (CSPC) or castration-resistant prostate cancer (CRPC).

Among PC cM1 patients, mean DKK-1 levels were significantly higher in patients with castration-sensitive (CS) tumors compared to those with castration-resistant (CR) PC (3591.4 pg/ml (SEM 852.0) versus 2262.1 pg/ml (SEM 371.7); *p* = 0.02; Figure 2). No associations between DKK-1 levels and the presence of lymph node (*p* = 0.27) or visceral (*p* = 0.13) metastases were detected in PC M1 patients. Also, there were no significant correlations between DKK-1 levels and patient age, T stage, Gleason score, or PSA level.

### 3.3. Sclerostin Serum Concentrations

**Figure 3:**
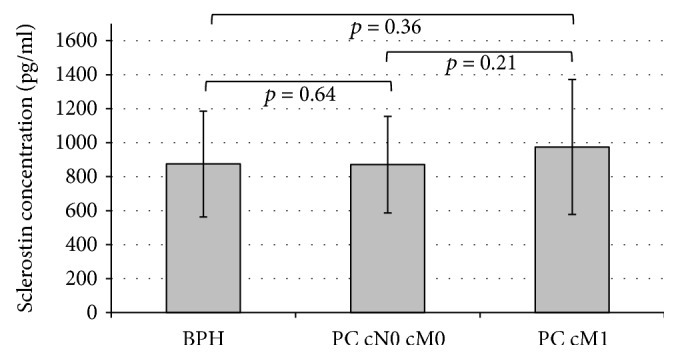
Mean sclerostin concentrations with standard error of the mean (SEM) in patients with BPH, prostate cancer without metastases (PC cM0), or prostate cancer with metastases (PC cM1).

Mean concentrations of serum sclerostin in patients with BPH, PC cM0, and PC cM1 were 874.4 pg/ml (SEM 42.7), 870.5 pg/ml (SEM 44.4), and 974.1 pg/ml (SEM 57.9), respectively. No significant differences in sclerostin concentrations were detected among these groups (Figure 3). Serum sclerostin levels showed a significant correlation with age (*p* = 0.02). Furthermore, within PC patients, sclerostin correlated significantly with total PSA serum concentrations (*p* = 0.01). In patients with PC cM0, sclerostin levels were not associated with features of adverse clinical outcome such as locally advanced disease, lymph node involvement, or Gleason score.

**Figure 4:**
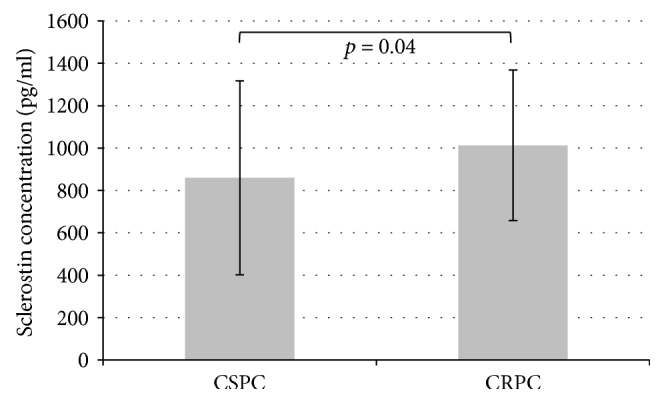
Mean sclerostin concentrations with standard error of the mean (SEM) in patients with castration-sensitive prostate cancer (CSPC) or castration-resistant prostate cancer (CRPC).

In the PC cM1 cohort, sclerostin levels were significantly higher in patients with CRPC than in patients with CSPC (1013.2 pg/ml (SEM 355.7) versus 859.7 pg/ml (SEM 457.7); *p* = 0.04; Figure 4). In PC cM1 patients, associations between sclerostin levels and the presence of lymph node (*p* = 0.39) or visceral metastases (*p* = 0.72) were not statistically significant.

## 4. Discussion

The Wnt pathway seems to be a central regulator of osteoblast function and bone development [10]. DKK-1 and sclerostin inhibit osteoblast activity by blocking Wnt pathway signaling and thereby contribute to progression of osteolytic metastases [4]. However, it is unknown whether these proteins are altered in patients with metastatic PC. Therefore, we investigated systemic alterations of DKK-1 and sclerostin levels in patients with BPH, clinically localized PC, and metastatic PC.

We observed significant differences in serum concentrations of DKK-1 between these three clinical groups. In accordance with the increased bone turnover found in metastatic PC, DKK-1 serum concentrations were significantly elevated in patients with bone metastases compared to localized PC [11]. So far, there is limited evidence to suggest that Wnt inhibitors (including DKK-1) are activated in tumors with osteoblastic features [12]. In this PC cohort, DKK-1 distinguished patients with and without metastasis. These findings are comparable to other data in which higher DKK-1 concentrations correlated with an increased aggressiveness of disease [7].

Investigating the subgroup of patients with metastatic PC, mean DKK-1 levels were significantly lower in patients with CRPC compared to CSPC. These data support the thesis of Hall et al., who proposed that level of Wnt inhibitors such as DKK-1 is a dynamic process that changes during disease progression [7]. Moreover, DKK-1 levels may also correlate with androgen receptor function and steroid signaling pathways [7]. Hence, antihormonal treatment-induced osteopenia in PC patients may have a significant impact on the Wnt pathway that all patients with PC M1b in this study were treated with antihormonal therapy confound interpretation of the presented findings. Therefore, further studies investigating Wnt inhibitors in patients treated or not treated with antihormonal therapy are needed.

In benign prostatic disease, serum levels of DKK-1 were higher than in PC patients without metastases. This finding may indicate that absence of Wnt pathway inhibition by DKK-1 promotes oncogenesis, an idea supported by previous data showing that modulators of bone turnover can be altered not only in metastatic disease but also during early stages of tumor progression [7, 13]. Liang et al. also observed lower median DKK-1 level in PC patients compared to controls [14]. However, this finding failed to meet statistical significance. Data regarding differences of DKK-1 in BPH and PC are scarce and partially discrepant [13]. Our findings, together with the lack of correlation between DKK-1 tissue expression and DKK-1 serum levels, may be explained by the hypothesis that PC tissue-derived DKK-1 only modestly influences systemic serum DKK-1 levels [13].

Therefore, it remains unclear whether tumor-associated DKK-1 expression is more substantial than systemic endogenous expression in bone tissue. The fact that DKK-1 expression in PC tissue is heterogeneous limits the diagnostic value of DKK-1 and may explain the lack of correlation between tumor and serum DKK-1 concentrations [15, 16].

Sclerostin seems to inhibit bone formation processes and stimulate bone resorption [17]. Without regard to the underlying disease, sclerostin concentrations are typically increased in patients with elevated bone turnover [17]. In our study, sclerostin concentrations were not significantly higher in patients with bone metastases compared to nonmetastatic PC. However, sclerostin levels correlated with serum total PSA concentrations in patients with PC. In contrast to DKK-1, sclerostin concentrations were significantly higher in patients with CRPC than in patients with CSPC. Similar findings were reported by other authors in PC patients with metastatic bone disease [17]. In this study, sclerostin levels were significantly higher in patients with advanced disease and increased bone turnover due to a compensatory response to the increased number of osteoblasts [17]. Also, the correlation between sclerostin and PSA might simply be due to the usually higher PSA levels seen in advanced PC disease. Even though sclerostin has been investigated in several bone diseases, published data from patients with different bone metabolism are rare. Apart from a possible pharmaceutical reason (antihormonal treatment affects sclerostin levels), age and sex also affect serum sclerostin levels [18]. We also noted that sclerostin levels correlated with age in this study. Although the physiological explanation for age-dependent increase remains unclear, increased sclerostin levels seen in males with increasing age indicate that patients are at risk for PC and increased osteocytic expression of sclerostin is expected. Other factors, such as bone density, physical constitution, or ethnicity, may also influence sclerostin production and the Wnt pathway [19].

Limitations of this study include the retrospective study design, the heterogeneous patient cohort, and the relatively small number of patients. Furthermore, the frequent use of antihormonal therapy and advanced age of the study population may compromise the interpretation of the results. In addition, the presence of other bone diseases that may have possibly influenced DKK1 and sclerostin was not assessed in this study.

Finally, the various aspects of the Wnt pathway in neoplastic disease have to be pointed out. In contrast to localized disease, metastatic tumor cells may interact locally with osteoblasts and osteoclasts, leading to changes in bone metabolism. Furthermore, a higher amount of DKK-1 may be produced by osteocytes compared to organ-confined tumors. As a consequence, DKK-1 should decrease in the presence of high-volume bone metastases. However, this is contradictive to our findings where higher DKK-1 levels were found in patient with PC M1b. One explanation might be possible switch in phenotype of different types of metastases. In addition, antihormonal treatment-induced osteopenia may also have a significant impact on DKK-1 and sclerostin levels. Regardless, it remains unclear whether any of these factors influence the Wnt pathway in this setting.

## 5. Conclusion

Wnt inhibitors seem to be involved in the progression of PC, especially with the development of bone metastases and CRPC. DKK-1-mediated inhibition of osteoblasts may play a role in the development of osteoblastic metastases. However, these data support the concept that DKK-1 and sclerostin levels in PC are heterogeneous processes which may vary during disease progression.

## Figures and Tables

**Table 1:** Characteristics of patients with prostate cancer without metastases (PC cM0) or with metastases (PC cM1).

	PC cM0	PC cM1
*N*	43	47
Age (median; range)	69 (48–85)	74 (52–84)
PSA, ng/ml (median; range)	6.96 (0.31–44)	80.4 (0.52–674)
Gleason Score, *n* (%)		—
≤6	7 (16.3)	—
7	31 (72.1)	1 (2.1)
≥8	5 (11.6)	4 (8.5)
T stage, *n* (%)		—
pT2a	6 (14.0)	—
pT2b	1 (2.3)	—
pT2c	24 (55.8)	—
pT3a	9 (20.9)	—
pT3b	3 (7.0)	—
N stage, *n* (%)		
N0	41 (95.3)	20 (42.5)
N+	2 (4.7)	25 (53.2)
Nx	—	2 (4.3)
M stage, *n* (%)		
cM1b	—	47 (100)
cM1c	—	8 (17.0)
Castrate level, *n* (%)		
CSPC	—	11 (23.4)
CRPC	—	34 (72.3)
n.a.		2 (4.3)

T stage = primary tumor; N stage = lymph nodes; M stage = distant metastases; N0 = no regional lymph node metastasis; N1 = metastasis in regional lymph node(s); Nx = regional lymph nodes were not assessed; cM1b = bone metastases; cM1c = other metastases; CSPC = castration-sensitive prostate cancer; CRPC = castration-resistant prostate cancer; n.a. = not applicable.

## Data Availability

The data (patient characteristics, serum levels, and measurement of DKK-1 and sclerostin and statistical analysis) used to support the findings of this study are available from the corresponding author upon request.
